# Connecting Openness and the Resting-State Brain Network: A Discover-Validate Approach

**DOI:** 10.3389/fnins.2018.00762

**Published:** 2018-10-22

**Authors:** Junjie Wang, Yang Hu, Hong Li, Ling Ge, Jing Li, Long Cheng, Zhi Yang, Xinian Zuo, Yong Xu

**Affiliations:** ^1^Department of Psychiatry, First Hospital/First Clinical Medical College of Shanxi Medical University, Taiyuan, China; ^2^Laboratory of Psychological Health and Imaging, Shanghai Mental Health Center, Shanghai Jiao Tong University Medical School, Shanghai, China; ^3^Institute of Psychology, Chinese Academy of Sciences, Beijing, China; ^4^Department of Medical Psychology, Shanxi Medical College of Continuing Education, Taiyuan, China; ^5^Multi-Disciplinary Team (MDT) Center for Cognitive Impairment and Sleep Disorders, First Hospital of Shanxi Medical University, Taiyuan, China; ^6^Key Laboratory of Cell Physiology in Shanxi Province, Taiyuan, China

**Keywords:** openness, parietal memory network, resting-state fMRI, data-driven, functional connectome

## Abstract

In personality neuroscience, the openness-brain association has been a topic of interest. Previous studies usually started from difference in openness trait and used it to infer brain functional activity characteristics, but no study has used a “brain-first” research strategy to explore that association based on more objective brain imaging data. In this study, we used a fully data-driven approach to discover and validate the association between openness and the resting-state brain network. We collected data of 120 subjects as a discovery sample and 56 subjects as a validation sample. The Neuroticism Extraversion Openness Five-Factor Inventory (NEO-FFI) was used to measure the personality characteristics of all the subjects. Using an exploratory approach based on independent component analysis of resting-state functional magnetic resonance imaging (fMRI) data, we identified a parietal network that consisted of the precuneus and inferior parietal lobe. The inter-subject similarity of the parietal memory network exhibited significant associations with openness trait, and this association was validated using the 56-subject independent sample. This finding connects the openness trait to the characteristics of a neural network and helps to understand the underlying biology of the openness trait.

## Introduction

On the basis that human experiences and behaviors are generated by biological processes, primarily within the brain, we seem to assume that the regularities in these experiences and behaviors that constitute personality are associated with regularities in the biological functions of the brain, making *personality neuroscience* possible (Canli, 2008; DeYoung and Gray, 2009). There have been a large body of studies aiming to test and refine neurobiological theories of personality by using neuroscientific methods such as molecular genetics (Malhotra et al., 1996; Kalbitzer et al., 2009), electroencepholography (EEG; Diethelm and Simons, 1945; Stough et al., 2001; Schmidtke and Heller, 2004), and positron emission tomography (PET; Johnson et al., 1999; O’Gorman et al., 2006). As a major non-invasive brain mapping technique, functional magnetic resonance imaging (fMRI) has relatively high spatial and temporal resolution. In recent years, the number of researchers using fMRI to explore *personality neuroscience* increased.

Constituted of five broad domains: Extraversion, Neuroticism, Agreeableness, Conscientiousness, and Openness, the Five Factor Model (FFM) is the most widely used descriptive taxonomy of personality and provides a common language for personality research (Costa and McCrae, 1992; John et al., 2008). There have been numerous studies that linking the individual variation in the structure (Omura et al., 2005; Rauch et al., 2005; Xu and Potenza, 2012) and function (Adelstein et al., 2011; Passamonti et al., 2015; Xiang et al., 2016) of different brain regions to taxonomies of FFM. We were particularly interested in the personality trait openness, which is a normally distributed personality trait reflecting the tendency to engage in imaginative, creative, and abstract cognitive processes. As one of the most important dimensions of personality traits, openness has always been one of the research hotspots of personality-brain association.

Previous studies of openness-brain associations usually started from the personality trait (openness) concept that is formed by observer-dependent life experience and consensus. It is doubtful whether the concept of personality, which is not completely independent of the subjective experience of observers, can fully and objectively reflect the activity characteristics of brain function networks. Besides, the subjective concept-based group has the assumption that all subjects in one group is homogeneous in brain mechanism, i.e., they share common intrinsic connectivity networks (ICNs). In fact, previous studies have found that personality traits were mostly associated with brain functional connections that were inconsistently present across participants (Di Martino et al., 2009; Adelstein et al., 2011).

In the current study, we adopted a data-driven approach, generalized ranking and averaging independent component analysis by reproducibility (gRAICAR; Yang et al., 2008; Yang et al., 2012, 2014a; Tian et al., 2018), to investigate whether the subjects can be grouped into communities according to the characteristics of their ICNs. The openness scores were not used to define subject groups prior to gRAICAR analysis, but they were associated to the ICN-derived subject communities to interpret the findings in neuroimaging data. With the effort, we attempted to identify association between ICNs and openness, aiming to generate objective and reliable metric of openness based on brain’s intrinsic functional activity characteristics.

**FIGURE 1:**
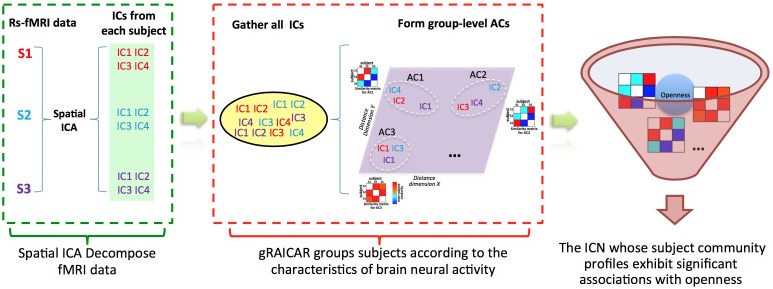
Demonstration of data analysis workflow. For simplicity, we assume that there are only three subjects (denoted as S1, S2, and S3). First, the fMRI data of subjects are decomposed individually by using spatial independent component analysis (ICA) into spatial components (ICs). Assume that for each subject we can get four ICs that are color coded to indicate which subject they are from. The resultant ICs maps are presented in the green layer. Second, all of the ICs from individual subjects were pooled in gRAICAR (as presented in the yellow layer). We present a distance space depicting the similarity between all ICs in the purple layer. The intention of gRAICAR in this part is to identify ICs that are from different individuals but are close to each other (as marked with white dashed circles). The group-level aligned components (ACs) were formed by these clustered ICs sequentially, and a community detection algorithm can be applied to each AC to identify homogeneous subject communities among all subjects. Third, we try to seek the intrinsic connectivity networks whose subject community profiles exhibit significant associations with openness.

We recruited 120 participants to investigate their characteristics of ICNs and associated the openness scores to the ICN-derived subject communities (Figure 1 shows a graphical demonstration of data analysis process). Furthermore, an independent sample of 56 subjects was recruited as a validation sample aiming to verify the findings from the discovery sample.

## Materials and Methods

### Participants

A total of 176 participants, including 120 participants as a discovery sample (age range: 19–60 years; 53 males, years of education: 14.49 ± 2.8, see Table 1 for details) and 56 participants as a validation sample (age range: 18–26 years; 12 males, years of education: 14.00 ± 2.5, see Table 1 for details) were recruited from Shanxi medical university or local communities. The inclusion criteria for the healthy participants were as follows: (1) age range from 18 to 60; (2) no serious physical diseases, pregnancy, or substance abuse; (3) no psychoactive substance use for at least 1 month; and (4) no history of mental disorder. The exclusion criteria for healthy subjects were as follows: (1) meet the criteria for any mental disorder according to DSM-IV; (2) family history of mental disorder; (3) history of taking antipsychotic drugs; and (4) unsuitability for MRI scans (metal implants or claustrophobia). The Ethics Committee at Shanxi Medical University approved the study protocol. Written informed consent was obtained from each participant prior to data acquisition. After the subjects completed the personality inventory and MRI scan, they subsequently received payment for their time. The methods were carried out in accordance with the approved guidelines.

**Table 1:** Descriptive statistics for the demographic characteristics of the two samples.

	Age range	Male	Female	Number	Percentage (%)
Sample (120)	18–20	0	2	2	1.67
	21–30	17	16	33	27.50
	31–40	10	10	20	16.67
	41–50	16	28	44	36.66
	51–60	10	11	21	17.50
Sample (56)	18–20	3	21	24	42.86
	21–26	9	23	32	57.14


### Behavior Data Acquisition

We used NEO-FFI (Neuroticism Extraversion Openness Five-Factor Inventory) to measure personality characteristics of all participants. NEO-FFI has been proven to be consistent with the full version, and it also has good convergent validity with other personality inventories (Costa and McCrae, 1989; Parker and Stumpf, 1998; Kurtz and Sherker, 2003).

### MRI Data Acquisition

All imaging data were collected using a 3.0 Tesla Siemens Trio MRI scanner at the Shanxi Provincial People’s Hospital. Resting-state scans were acquired with an echo-planar imaging (EPI) sequence (32 axial interleaved slices, acquired from inferior to superior, TR/TE = 2,500/30 ms, FA = 90°, FOV = 240 mm, matrix = 64 × 64, thickness = 4.0 mm, gap = 0.0 mm, 212 volumes, duration 8′50^′′^). High-resolution anatomical scans were acquired with a T1-weighted 3D MP-RAGE sequence (TR/TE/TI = 2,300/2.95/900 ms, FA = 9°, slices = 160, thickness = 1.2 mm, FOV = 225 mm × 240 mm, matrix = 240 × 256).

### Image Preprocessing and Quality Control

Both anatomical and resting-state fMRI images were performed using the Connectome Computation System (Zuo et al., 2013), an integrated data preprocessing system that connects AFNI, FSL, and Freesurfer (Cox, 2012; Fischl, 2012; Jenkinson et al., 2012). Structural images were first cleaned by using a spatially adaptive non-local mean filter to remove noise (Zuo and Xing, 2011) and fed into FreeSurfer 5.1 for extracting the brain as well as for segmenting the brain tissues into gray matter, white matter and cerebrospinal fluid. All images were converted into MNI152 space using Advanced Normalization Tools (ANTs; Avants and Gee, 2004).

The following preprocessing steps were applied to the resting-state fMRI images: (1) the first five volumes were discarded to allow MRI signal equilibration; (2) slice timing differences were corrected; (3) the head movements were realigned over the entire scan; (4) the mean resting-state fMRI image was spatially normalized to MNI152 space via the combined registration of a rigid transformation of the individual structural images and nonlinear ANTs transformation; (5) the 4D data were standardized to a global mean intensity of 10,000; (6) the data were temporally band-pass (0.01–0.1 Hz) filtered.

The quality control procedure of anatomical images was performed by two researchers, including visual inspections of quality of tissue segmentation and head motion. For functional images, subjects would be excluded for excessive head motion evaluated using mean frame-wise displacement (meanFD), so that the meanFD was less than 0.2 mm.

### gRAICAR Network Mining Analysis

Generalized ranking and averaging independent component analysis by reproducibility was applied to the preprocessed functional images for the purpose of characterizing the inter-subject similarity of the ICNs. By using gRAICAR, we could know how strong the one-to-one correspondence is, subsequently, we could reveal variations of brain maps across different subjects. Briefly, for each subject, the data were decomposed into spatial independent components (ICs) using the MELODIC module of FSL (Beckmann and Smith, 2004), and the spatial maps of the components were transformed into the MNI152 space. gRAICAR then pooled all of the ICs from each subject and matches them across all subjects to form a set of group-level aligned components (ACs). For each of the ACs, a spatial similarity matrix was computed to reflect the similarity between its comprising ICs, each representing a subject. This inter-subject similarity matrix represented a subject community profile that reflected potential subgroups of subjects sharing similar ICN characteristics.

To examine the significance of the associations between the inter-subject similarity derived from the ACs and the individual differences in the openness scores, we conducted a permutation test. First, each row of the inter-subject similarity matrix for each AC were summed to yield a degree of centrality for each subject in the given AC. The subjects were then classified into high openness score group (HOS, *n* = 83) and low openness score group (LOS, *n* = 37) according to the division standard of NEO-FFI. The between-group difference of the similarity degree was calculated. Under the null hypothesis that there was no significant difference in the similarity degree between the HOS and LOS subjects, the group identities of the subjects were then randomly permuted 8,000 times to yield a null distribution of the between-group difference of the similarity degrees. The percentile of the original between-group difference in the similarity degree indicated whether there was a significant association between the AC-derived inter-subject similarity and the individual difference in the openness classes.

## Results

### Demographical and Behavioral Measures

The demographical information and descriptive statistics for the NEO-FFI scores of all subjects in both experimental sample and validation sample are shown in Tables 1 and 2. There was no significant difference between the two samples in terms of Openness scores (*t* = -1.879, *p* = 0.06), Extraversion scores (*t* = 1.027, *p* = 0.30) and Agreeableness scores (*t* = -1.424, *p* = 0.15). There was significant difference between the two samples in terms of Neuroticism scores (*t* = 2.173, *p* = 0.03) and Conscientiousness scores (*t* = -3.728, *p* < 0.01).

**Table 2:** Descriptive statistics for the NEO-FFI scores of all participants in the two samples.

Dimension of NEO-FFI	Mean (120)/Mean (56)	SD (120)/SD (56)	Range (120)/Range (56)
Neuroticism	31.93/34.14	6.25/6.42	16–39/21–49
Extraversion	39.64/40.71	6.67/5.96	20–56/28–53
Openness	38.90/37.79	4.53/3.18	28–53/30–43
Agreeableness	42.00/41.13	3.78/3.84	31–51/34–49
Conscientiousness	42.18/38.64	6.05/5.45	24–58/26–52


### gRAICAR Findings

The gRAICAR algorithm identified thirty ACs. Based on previous literature (Beckmann et al., 2005; Damoiseaux et al., 2006, 2008) and their spatial patterns, 12 ACs were found to represent functional ICNs. The remaining 18 components reflected artifacts like cerebrospinal fluid flow, physiological noise, and movement.

The inter-subject similarity matrix for each of the 12 ICNs reflected a subject community profile which reflect potential subgroups of subjects that share similar ICN characteristics. Thus, we explored the associations between the personality scores and the similarity matrix. We reordered the matrix according to hypothesized groupings and check whether the reorder the inter-subject similarity matrix exhibits a clear cut-off between groups. By visual inspection, we found three ICNs that may be associated with three personality dimensions. However, after the analysis of voxel-wise functional connectivity strength, the Salience Network-Extroversion association (*t* = -0.25, *p =* 0.801) and the Sensorimotor Network-Neuroticism association (*t* = 1.43, *p =* 0.156) were not significant and therefore were excluded. In the following sections, we report remaining one ICN that were related to NEO-FFI-Openness classification.

### An ICN Associated With Openness Groups

**FIGURE 2:**
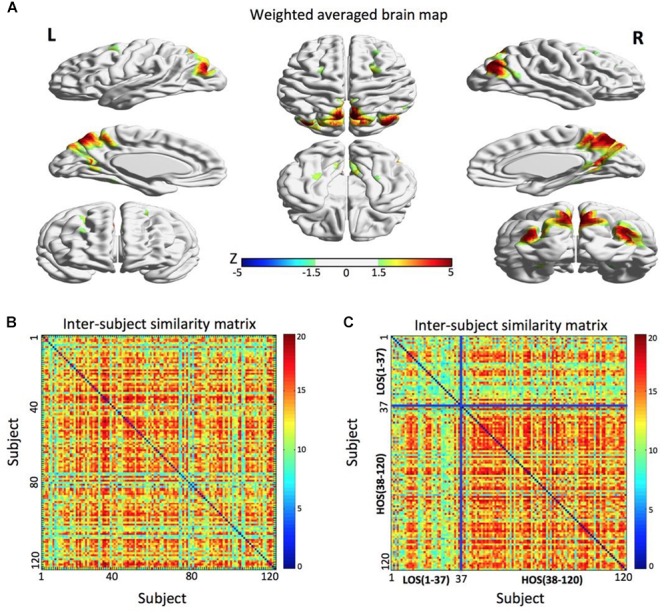
The parietal memory network is associated with openness groups. **(A)** The parietal memory network rendered onto cortical surface of the brain. The network consists of three brain regions located in the precuneus, left inferior parietal lobule, and right inferior parietal lobule. The maps were thresholded at | *Z*| > 1.5 for better visualization on the surfaces. **(B)** The similarity matrix of the parietal memory network across 120 subjects has no regular distribution. Both horizontal and vertical axes represent subjects. **(C)** Combined with the openness scores, the similarity matrix change into a regular distribution. Compared to the LOS subjects, the HOS subjects have a higher inter-subjects average similarity. For visualization purpose, the subjects are grouped into LOS and HOS groups, and the blue solid lines mark the boundary between the two groups. All surface maps are rendered in BrainNet Viewer (Xia et al., 2013).

A parietal memory network (PMN, Gilmore et al., 2015) formed by the precuneus (PCU; *x* = 6 mm, *y* = -69 mm, *z* = 60 mm in MNI space) and left and right inferior parietal lobule (l-IPL; *x* = -39 mm, *y* = -84 mm, *z* = 27 mm and r-IPL; *x* = 42 mm, *y* = -78 mm, *z* = 33 mm) reflected a subject community profile that was associated with the openness classification (Figure 2). The inter-subject similarity matrix of this network depicted similarities among all of the subjects.

After the permutation test, we found that there was a significant difference in the average similarity degree between the HOS subjects and the LOS subjects (*p* < 0.01). Visually, we found that the original inter-subject similarity matrix (Figure 2B) showed a clearer block when the subjects were reordered according the openness score (Figure 2C), with the block representing the subjects having highly similar ICN. These findings suggest that the PMN can distinguish the HOS subjects from the LOS subjects.

### Analysis of Voxel-Wise Functional Connectivity Strength

The gRAICAR results revealed that the openness groups were associated with the inter-subject similarity reflected in the PMN. In order to provide supporting evidence for the gRAICAR findings from an independent methodology, we performed *post hoc* analyses to examine functional connectivity strength between the regions in the identified PMN and its associations with openness groups.

**FIGURE 3:**
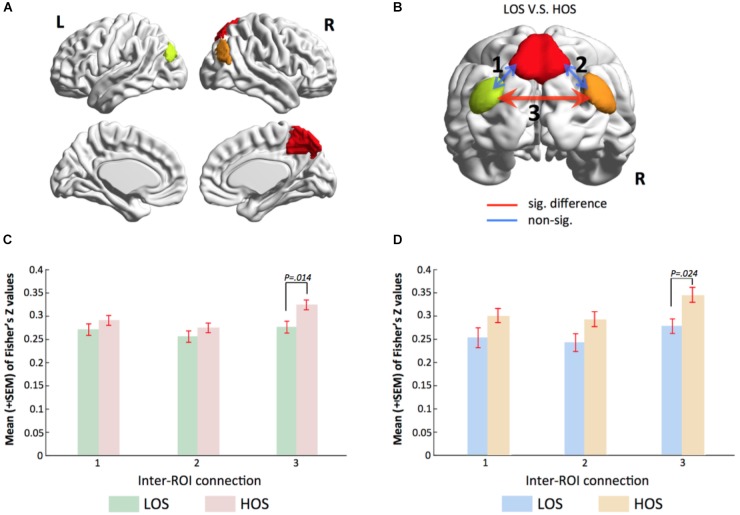
Voxel-wise functional connectivity analyses validate gRAICAR findings. **(A)** Three regions of interest including the PCU, the l-IPL and the r-IPL. **(B)** Map of the parietal memory network showing inter-regional connections exhibiting significant (red line) and non-significant (blue lines) differences in connectivity strength (Fisher’s *Z*) between LOS and HOS groups. **(C,D)** Bar graphs showing statistical results in comparing functional connectivity strength between LOS and HOS groups. **(C)** presents the results from the discovery sample and **(D)** from validation sample. Labels along the horizontal axis correspond to the connections marked on **(B)**. In both the discovery data and the validation samples, the connectivity between bilateral IPL shows significant difference between LOS and HOS groups.

By applying a threshold of *Z* > 3.5 and a cluster size >30 voxels to the brain map of the PMN, we obtained three regions of interests (ROIs), including the PCU, the l-IPL, and the r-IPL (Figure 3A). For each subject, the time series at each voxel within these ROIs was extracted to construct a voxel-wise correlation matrix. The correlation coefficients between voxels belonging to different ROIs were converted into Fisher’s *Z* values and then averaged to obtain a metric of inter-ROI functional connectivity for each connection. A network model highlighting the functional connectivity difference between the HOS and LOS subjects is shown in Figure 3B and the corresponding statistical comparisons are displayed in Figure 3C. One-way ANCOVA with age, sex, years of education, and meanFD as covariates was performed to examine the differences in inter-ROI functional connectivity between HOS and LOS subjects.

The results showed higher inter-ROI functional connectivity in HOS than in LOS subjects in connections between the l-IPL and the r-IPL (connection 3 in Figure 3B, *F* = 6.216, *p* = 0.014).

### Independent Validation of the Findings

In order to verify our findings in an independent sample, we used the same ROIs to conduct a functional connectivity analysis in the validation sample. Consist with our previous findings, the results revealed that functional connectivity strength between bilateral IPL in the HOS subjects is higher than that in the LOS subjects (connection 3 in Figure 3B, *F* = 5.421, *p* = 0.024) and the corresponding statistical comparisons are shown in Figure 3D.

### Correlations Between Connectivity Strength in PMN and Openness Scores

**FIGURE 4:**
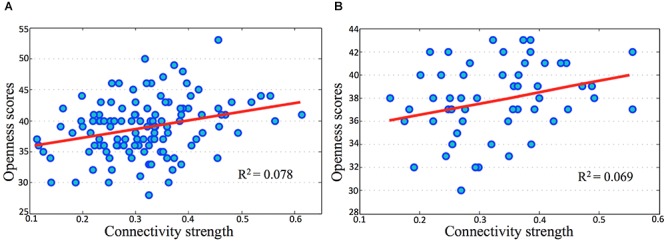
Correlations between openness scores and connectivity strength in parietal memory network. The horizontal axes present connectivity strength (Fisher’s *Z*) between the l-IPL and the r-IPL, and the vertical axes present the openness scores. **(A)** discovery sample, age and gender were controlled, *r* = 0.278, *p* < 0.01; **(B)** validation sample, age and gender were controlled, *r* = 0.277, *p* < 0.05.

In both the discovery and validation samples, openness scores significantly correlated with connectivity strength (Fisher’s *Z*) between the l-IPL and the r-IPL (Figure 4). We conducted a hierarchical regression analysis and the result indicated that the predictor of openness scores was connectivity strength between the l-IPL and the r-IPL. In the discovery sample, the predictor accounted for 7.8% of the model variance after controlling for age (*adjusted R*^2^ = 0.078, *F* = 11.118, *p* < 0.01). In the validation sample, the predictor accounted for 6.9% of the model variance after controlling for age (*adjusted R*^2^ = 0.069, *F* = 5.049, *p* < 0.05).

## Discussion

In this study, following a brain-first research strategy, we applied a discover-validate approach to investigate the characteristics of brain intrinsic networks with a sample of 120 healthy participants and found a reliable association between the PMN and NEO-FFI-Openness, which was confirmed in a subsequent validation study with a sample of 56 participants. Specifically, the results showed higher inter-ROI functional connectivity in HOS than in LOS subjects in connections between the l-IPL and the r-IPL, and that people who have a higher functional integration of PMN exhibited a higher score on the NEO-FFI-Openness trait.

McCrae and Costa have pointed out that HOS people have a stronger information processing ability, so that the breadth and depth of their cognition has increased (McCrae and Costa, 1997). Furthermore, in the FFM, openness is the only one trait that has a positive correlation with intelligence (Gray et al., 2003; DeYoung et al., 2005). As an important multimodal hubs in the brain for both structure and functional connectivity (Hagmann et al., 2008; Sporns, 2011; van den Heuvel and Sporns, 2011), PCU and IPL play important roles in the process of individual cognition (Kjaer et al., 2002; Lou et al., 2004; vogt and Laureys, 2005; Andrews-Hanna et al., 2010; Gilmore et al., 2015). Moreover, they have been closely related to the intelligence level of the individual (Owen et al., 2005; Jung and Haier, 2007). There has been a contention that openness may be related to the default mode network (DMN) functioning since both openness and the DMN are associated with imaginative cognition (DeYoung, 2014, 2015). Some evidence from personality neuroscience suggested that there is a stable positive correlation between openness and individual regions of the DMN (Raichle et al., 2001; Greicius et al., 2003; DeYoung et al., 2010; Adelstein et al., 2011; Beaty et al., 2016). The current study refined this argument to the PCU and IPL.

Changes in the PCU, which have been considered to play an essential role in individual consciousness (Cavanna, 2007), have previously been reported to be relevant to schizophrenia (Buckner et al., 2008; Whitfield-Gabrieli et al., 2009; Kühn and Gallinat, 2013). One of our previous studies (Yang et al., 2014b) adopting the same neuroimaging data-mining approach used in the present study, gRAICAR, found that the PCU and bilateral angular gyri (AG, part of the IPL) were absent in early-onset schizophrenia patients, and the results implied that the PCU-AG network is one of the primary targets affected by schizophrenia. Given the previous findings that patients with schizophrenia showed lower scores for openness compared with healthy subjects (Ohi et al., 2016), our findings that the PMN were statistically absent in LOS subjects can partly support the viewpoint that personality is one of important factors in the pathogenesis of schizophrenia since it affects subjects’ cognition and social functioning as well as the patients’ clinical symptoms (Ohi et al., 2016).

Yang et al. (2016) used a classical twin study design to examine the heritability of intrinsic functional network properties in 101 twin pairs, in which they showed that the activity of PMN is strongly heritable. Given our findings of a reliable association between the PMN and openness scores, it is reasonable to speculate a relationship between openness and genetics. In fact, twin studies have shown that personality traits are moderately heritable (Munafo et al., 2008) and have a relatively stable trajectory over time after early adulthood (Kupper et al., 2011). In addition, Amin et al. (2013) conducted a meta-analysis of four genome-wide linkage scans and identified 11q24 for openness to experience (NEO). By conducting a meta-analysis of NEO personality traits, de Moor et al. (2012) found significant associations for openness near the gene RASA1.

In the current study, we adopted a brain-first research strategy, trying to establish a hypothesis based on the differences of inter-individual brain intrinsic networks characteristics, and then inferred the personality trait (openness) associated with a certain brain intrinsic network characteristic. Psychologist has been observing the psychological world using categories derived from our own experiences, naming these categories using common sense words and searching for the counterparts of these categories within the brain. Since there has been a debate that these observer-dependent categories may not directly correspond (in a one-to-one fashion) to the observer-independent facts of the brain mechanism, thus, psychology may need a different set of psychological categories that more closely reflect the brain’s activities in creating our mind and causing our behavior (Barrett, 2009).

Personality traits can be closely aligned with domains within the ‘Research Domain Criteria’ project (RDoC; Insel et al., 2010; Insel, 2014; Simmons and Quinn, 2014), which views psychiatric disorders as extremes of normal tendencies, and is intended to foster a biological analysis of behavior (Sanchez-Roige et al., 2018). While numerous fMRI studies aiming to establish the mental disorders classification system based on brain mechanism have examined psychiatric diseases (Fair et al., 2012, 2013; Yang et al., 2014b), relatively less work has been done on the brain-first basis of RDoC traits such as personality. In addition, some new associations between brain networks and clinical symptoms could only be detected in studies adopted RDoC strategy.

## Limitations and Future Directions

Combining findings of our research group in previous and the current work, it can be inferred that openness may be related to schizophrenia based on the characteristics of individual brain images. One of the prominent features of schizophrenia patients is impaired cognitive ability. Unfortunately, we did not have a systematic cognitive ability evaluations for our subjects. In future studies, relationships between ICNs, personality, and multi-dimensional cognitive abilities should be investigated.

As a study adopting the data-driven method, focusing only on differences in individual brain imaging features by fMRI is not comprehensive. The lower time resolution is a technically insurmountable defect. Future studies can collect higher time-spatial resolution data by using EEG-fMRI. Genetic information, if conditions permit, should also be collect with the aim of establishing a more comprehensive research about biological basis of openness based on a gene-brain-behavior holistic perspective.

## Author Contributions

YX and ZY designed and supervised the study. JW, YH, and HL drafted the manuscript. LG, JL, and LC carried out the experimental procedures. XZ undertook the statistical analyses and reviewed the literature.

## Conflict of Interest Statement

The authors declare that the research was conducted in the absence of any commercial or financial relationships that could be construed as a potential conflict of interest.
